# Matrix Optical Biosensor for Determining YKL-40/CHI3L1—A Biomarker Potentially Associated with Alzheimer’s Disease

**DOI:** 10.3390/bios15100687

**Published:** 2025-10-10

**Authors:** Zuzanna Zielinska, Abdulelah Ba Tarfi, Ewa Gorodkiewicz

**Affiliations:** 1 Faculty of Chemistry, Doctoral School of University of Białystok, University of Bialystok, Ciolkowskiego 1K, 15-245 Bialystok, Poland; 2 Bioanalysis Laboratory, Faculty of Chemistry, University of Bialystok, Ciolkowskiego 1K, 15-245 Bialystok, Poland; 3 Department of Chemistry and Bioengineering, Faculty of Fundamental Sciences, Vilnius Gediminas Technical University, Sauletekio al. 11, Vilnius, 10221 Vilniaus m. sav., Lithuania; abdobatarfi@gmail.com

**Keywords:** YKL-40, biomarker, neurodegenerative diseases, SPRi biosensor

## Abstract

YKL-40 is a glycoprotein that may be present at elevated levels in many cancers and neurodegenerative diseases. It has been investigated in numerous studies as a potential biomarker for several conditions, including Alzheimer’s Disease (AD). In this study, a biosensor with Surface Plasmon Resonance imaging (SPRi) detection, sensitive to YKL-40, was constructed for the detection of this analyte in the blood plasma of AD patients. Extensive validation of the biosensor was performed. This included the determination of analytical parameters such as the biosensor’s response characteristics, detection and quantification limits, precision, accuracy, repeatability, selectivity, stability, and performance in natural samples. Validation parameters were primarily tested using standard solutions, while natural samples were employed to evaluate repeatability, stability, and assay accuracy in three groups of samples from different patients. A YKL-40-specific antibody was used as the receptor layer, immobilized on a gold plate using the EDC/NHS protocol on thiol 11-MUA. The biosensor exhibited a wide operating range (1–200 ng/mL), a low detection limit (LOD) of 2 pg/mL, and a quantification limit (LOQ) of 7 pg/mL. High precision and accuracy were confirmed by the calculated standard deviations (SD) and coefficients of variation (CV), which ranged from 0.0009 to 7.02 ng/mL and from 0.12% to 9.24%, respectively. The sensor also demonstrated good repeatability (CV = 4.995%) and was capable of detecting the analyte of interest in complex biological matrices. Its applicability was confirmed in a study using plasma from AD patients and two selected control groups: plasma from smokers and patients with prostatitis. This allowed the assessment of YKL-40 levels across different groups. The results were consistent with literature values, and statistical analysis confirmed the significance of concentration differences between groups. Furthermore, ROC curve analysis confirmed the diagnostic usefulness of the constructed YKL-40 test in the context of Alzheimer’s disease.

## 1. Introduction

YKL-40, with a molecular mass of approximately 40 kDa, belongs to the group of glycoproteins that bind heparin and chitin. It is also known as chitinase-3-like protein 1 (CHI3L1). Other commonly used names include heparin-binding glycoprotein of 38 kDa and human cartilage glycoprotein-39 (HC gp-39) [1]. YKL-40 is secreted in vitro by various cells, including immune cells such as macrophages and neutrophils [2], as well as connective tissue cells, such as vascular smooth muscle cells or fibroblast-like synovial cells [3]. Its role is mainly discussed in the context of inflammation, tissue remodeling, and angiogenesis [1].

YKL-40 is involved in the development of the human musculoskeletal system, with both mRNA expression and protein secretion detected across all germ layers. Furthermore, it contributes to tissue proliferation, differentiation, and morphogenesis [4]. Research suggests that YKL-40 serves as a diagnostic, activity, and prognostic marker in several autoimmune diseases, including rheumatoid arthritis [5], psoriasis [6], and lupus [7]. Elevated YKL-40 levels have also been linked to the progression of multiple cancers, such as glioma [8], as well as gastric, colon, and gallbladder cancer [9]. YKL-40 secreted by macrophages may promote cell migration, for example, in breast cancer [10]. Clinical studies confirm the correlations between YKL-40 concentrations and the development and metastasis of various cancers [11]. In addition, it can contribute to cancer cells’ transformation, underscoring its potential as both a diagnostic biomarker and a therapeutic target [9]. As previously mentioned, glycoprotein YKL-40 is associated with inflammatory responses [2]. Its high expression may be observed in brain cells and astrocytes under neuroinflammatory states [12]. Literature reports indicate that chronic inflammation is a hallmark of Alzheimer’s disease (AD) [13]. The primary mediators of inflammation in AD are astrocytes and microglia. Chronic inflammation in AD is caused by the presence of intracellular neurofibrillary tangles (NFTs) composed of various forms of phosphorylated tau and beta-amyloid deposits [14], and by the activation of the neuronal death cascade [15]. Consequently, the brains of AD patients contain pathological structures consisting of fibrillar agglomerates of antibody fragments surrounded by microglia. These cells are identified as the primary mediators of the immune response, secreting proinflammatory cytokines [2]. Other reports indicate that YKL-40 may be elevated in various neurological disorders, including stroke, lentivirus encephalitis, traumatic brain injury, amyotrophic lateral sclerosis, and multiple sclerosis [16,17,18,19]. Due to its involvement in many disease processes, it is not considered a specific potential biomarker. However, when combined with other biomarkers, it can provide valuable additional information [20]. Several studies have examined plasma YKL-40 in AD. For example, M. P. Pase et al. investigated the usefulness of plasma YKL-40 as a biomarker of brain aging and dementia risk [21]. Their results indicated that higher plasma YKL-40 concentrations were associated with reduced brain volume and declining cognitive function in a study of over 6,000 participants. Furthermore, higher levels of this glycoprotein are associated with markers of brain health and with the risk of future dementia in adults without dementia. Therefore, YKL-40 is a promising blood biomarker that is useful in assessing the impact of inflammation on patients’ brains [21].

Cerebrospinal fluid (CSF) studies further demonstrate that YKL-40 levels are significantly higher in AD patients compared with controls, and increase with early disease progression [22]. Importantly, YKL-40 levels also rise during the preclinical and prodromal stages of AD, with higher concentrations associated with faster cognitive decline and an increased likelihood of progression to Alzheimer’s dementia. Thus, YKL-40 may support early diagnosis and monitoring of disease progression, particularly when combined with established biomarkers such as Aβ-42, total tau, and phosphorylated tau. Measuring YKL-40 also provides insights into astroglial activation and may enhance the diagnostic value of biomarker panels in clinical practice. Moreover, monitoring its levels may help evaluate the effectiveness of therapeutic interventions aimed at modulating the inflammatory response [22].

In addition, YKL-40 is a studied and tested glycoprotein with broad diagnostic potential; biosensors are being developed to detect it in tested samples. In a research experiment by W. Chaocharoen’s team, an electrochemical biosensor for detecting YKL-40 was fabricated [23]. While electroanalysis has not yet been widely applied in clinical biochemical analysis, it is nevertheless feasible, and the authors indicate that the sensor’s detection limit is approximately 300 times lower than that of ELISA. This method utilizes gold working electrodes with immobilized specific antibodies. These antibodies are covalently bound to the electrode surface using a thiol. Furthermore, a flow-injection analysis system is employed, which has been shown to significantly improve efficiency [23].

The literature also includes a report on a photothermal immunosensing platform for sensitive YKL-40 screening [24]. This approach combines platinum nanoparticles (PtNPs) excited with near-infrared (NIR) light and a handheld digital thermometer. Under optimal conditions, the system achieved a dynamic range of 0.03–100 ng/mL with a detection limit of 0.014 ng/mL. YKL-40 concentrations were successfully determined in blood serum samples using this method, and the results were also compared to those of ELISA. The sensor is characterized by high sensitivity and reproducibility [24].

In another study, S. Naglot’s research team applied SPR technology to study YKL-40, but they used the commercially available BIAcore 2000 device. These studies, performed on blood serum samples from patients with bronchial asthma, achieved a detection limit of 0.33 ng/mL [25].

This paper presents an SPRi biosensor for YKL-40 determinations in biological materials. There are no previous reports describing a biosensor of this kind. The above-mentioned reports describe, for example, an electrochemical immunosensor that, despite its high sensitivity, is unsuitable for real-time imaging and multiplexing [23]. The same applies to the photothermal platform [24], which requires labeling. In turn, the BIAcore 2000 does not support multi-site imaging or detailed spatial resolution [25]. The SPRi detection method used in this article offers significantly more possibilities. First, we do not need labeled receptor layers or complex components in this range to capture the analyte of interest from the sample. The method is based on a direct protein–antibody reaction.

Furthermore, the instrument is easy to use, and data acquisition and analysis are straightforward, significantly reducing the time needed to obtain results for biological material. The measurement itself, including receptor layer preparation, takes about an hour, and during this time, nine or even twelve samples can be labeled in a single measurement cycle. This is an additional advantage of our method. The innovative nature of SPRi is also manifested in its multiplexing capabilities. As mentioned earlier, YKL-40 is not a specific marker for AD. Nevertheless, its combination with other biomarkers, such as total and phosphorylated tau proteins, makes it a marker of high diagnostic utility. This report, concerning an SPRi biosensor for selective YKL-40 detection, is part of a project to construct a diagnostic panel for Alzheimer’s disease. A previous paper described a biosensor sensitive to erythropoietin [26]. This means that YKL-40 will not be considered a single marker but a complementary element, enhancing diagnostic power when combined with characteristic biomarkers of AD and potential markers such as the aforementioned erythropoietin. SPRi biosensors exploit the polarization of surface plasmons, i.e., electrons and their associated waves propagating on thin noble metal surfaces [27]. In the SPRi system used, a plate with a thin gold layer (50 nm) is arranged on the glass prism of the device, which is based on the Kretschmann configuration [28]. A monochromatic and p-polarized light beam, emitted by a laser, illuminates the gold layer at a specific angle, exciting the SPR, and inducing plasmon resonance. The reflected light is collected using a CCD camera [27]. At the metal–dielectric interface, the beam energy is coupled to plasmons, producing the SPR effect and reducing total internal reflection. SPR sensor components include an optical device, a processing medium combining biological and optical elements, and an electronic device that supports optoelectronics and enables data analysis [29]. Adding and immobilizing subsequent layers alters the reflection angle and the SPR effect, consequently changing the refractive index. This enables concentration-dependent analysis of the tested biomolecules. In SPRi, the entire surface of the measurement plate is displayed, allowing simultaneous monitoring of various interactions of the immobilized molecular layers [30]. Studying these interactions using SPR-based technologies allows real-time observations without labeling [31]. Surface Plasmon Resonance has enabled the development of biosensors capable of detecting numerous biomolecules, even those with low molecular weights. Therefore, it is suitable for applications in the pharmaceutical sciences, diagnostics, environmental and water monitoring, and product safety [32].

Improved biosensor technologies now offer disease detection through the determination of disease-specific biomarkers, and also enable monitoring of the body’s response to treatment [33]. YKL-40, a potential biomarker for various diseases, exhibits varying levels, which may depend on the condition [34]. In work by R. Craig-Schapiro et al., biomarkers used to detect early stages of Alzheimer’s disease were investigated [35]. YKL-40 was measured in both plasma and cerebrospinal fluid. Patients were analyzed according to the Clinical Dementia Rating (CDR) scale, a commonly used tool for assessing and staging dementia, including Alzheimer’s. It was found that the concentrations correlated with CSF levels. The values recorded were 91.9 ng/mL and 81.1 ng/mL in the AD group (CDR1 and CDR0.5, respectively) and 62.5 ng/mL in the control group (CDR0). The control group consisted of elderly people in good general health, having no other neurological, psychiatric, or major medical diagnoses that could contribute significantly to dementia, or use of exclusionary medications [35].

In another study by a team led by J. Choi, YKL-40 was investigated as a potential biomarker of AD. An increase in the mean plasma YKL-40 level was observed in AD patients (376.86 ng/mL) compared with the control groups—healthy older people (96.91 ng/mL) and MCI patients (176.49 ng/mL). The levels of the potential biomarker positively correlated with neuropsychological test results in MCI and AD patients. This confirms this biomarker’s diagnostic usefulness in analyzing Alzheimer’s disease [36].

## 2. Materials and Methods

### 2.1. Reagents

The biosensor was constructed on a base consisting of plates with a gold layer (Ssens, Enschede, The Netherlands). The following reagents were also used for the tests: thiol 11-mercaptoundecanoic acid (SIGMA, Steinheim, Germany), EDC (N-ethyl-N′-(3-dimethylaminopropyl)carbodiimide hydrochloride) (SIGMA, Steinheim, Germany), NHS N-hydroxysuccinimide (Aldrich, Munich, Germany), buffered saline solution (PBS buffer) (Biomed, Lublin, Poland), absolute ethyl alcohol (POCh, Gliwice, Poland), and ethanolamine solution (SIGMA, Steinheim, Germanyoo), recombinant human Chitinase 3-like 1 (YKL-40) protein (R&D Systems, Minneapolis, MN, USA), anti-hChitanase 3-like 1 (YKL-40) antibody (R&D Systems, Minneapolis, MN, USA), recombinant human HIF-1 alpha protein (Abcam, Cambridge, UK), recombinant human (phospho)-181 Tau protein (Abcam, Cambridge, UK), recombinant human ANG-2 protein (R&D Systems, Minneapolis, MN, USA), recombinant human IL-1 beta protein (R&D Systems, Minneapolis, MN, USA) and recombinant human Tau protein (R&D Systems, Minneapolis, MN, USA).

### 2.2. Biological Material

The biological material consisted of 18 plasma samples from patients diagnosed with Alzheimer’s disease, without comorbidities. The relevant Bioethics Committee of the Medical University of Białystok approved the study using this material (approval no. APK.002.596.2024, 20 March 2025). Additionally, 18 plasma control samples were used from patients who were not affected by Alzheimer’s disease but were smokers, as smoking causes hypoxia in the brain and can consequently lead to neurodegenerative conditions. Furthermore, smoking can cause chronic inflammation, especially in the lungs and circulatory system. In this group, we can determine whether YKL-40 exhibits elevated concentrations in nonspecific inflammatory processes or only in neurodegenerative pathology. The samples came from the Biobank of the Medical University of Białystok, and consent to conduct the study was obtained from the relevant bioethics committee (approval R-I-002/600/2019, 19 December 2019). Also, 18 plasma samples from patients with prostatitis were used as a control group. The research protocol received approval from the Bioethics Committee at the Medical University of Bialystok (approval number APK.002.307.2023, 22 June 2023). Prostatitis is a disease unrelated to the nervous system. It is known that YKL-40 may exhibit greater activity in case of inflammatory diseases. Assessing YKL-40 in this group enabled us to determine whether YKL-40 is elevated generally in inflammation or more specifically in CNS. The study was age- and gender-adjusted.

### 2.3. SPRi Instrumentation

**Figure 1:**
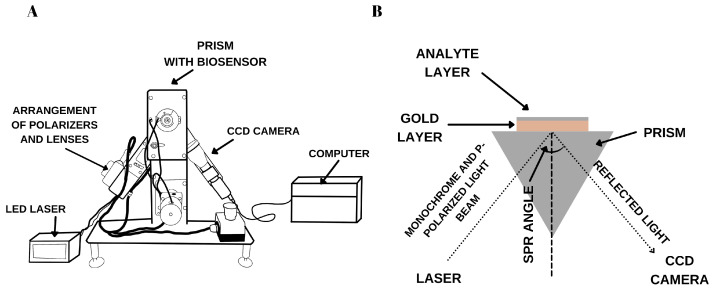
A diagram showing the construction of (**A**) the SPRi device with its components, (**B**) Kretschmann configuration.

The SPRi apparatus is a prototype developed at the Bioanalysis Laboratory, Faculty of Chemistry, University of Bialystok. The device components are shown in Figure 1A. The system includes a laser module that emits light at a wavelength of 635 nm, passing through a system of polarizers and lenses, and then striking a prism with a biosensor mounted on the device. Subsequently, the light is reflected from a thin metal surface in a Kretschmann configuration (Figure 1B). The reflected light is captured by a camera and transmitted to a computer for analysis. Using ImageJ software (version 1.53, National Institutes of Health, NIH), a quantitative signal is obtained after converting images into numbers using appropriate mathematical operations. The angle at which the desired minimum—that is, the best SPRi signal—occurs is selected using the device’s movable arms. This is the angle where the maximum contrast between the background and the sensor’s active areas is achieved. The analytical signal is defined as the difference in the intensity of the light reflected before and after interaction with the analyte.

### 2.4. Sensor Design

**Figure 2:**
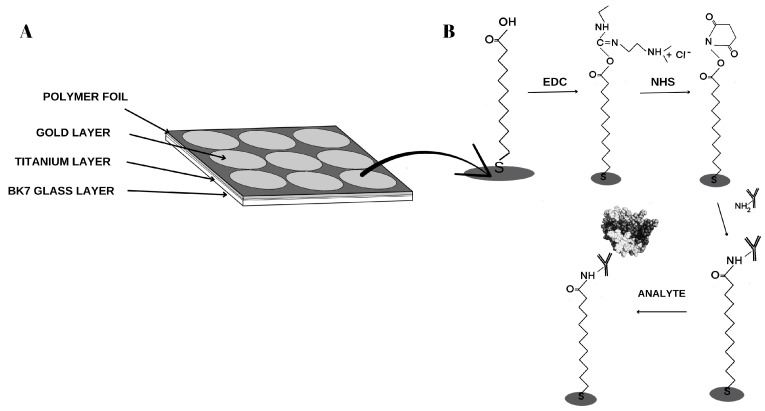
A diagram showing the construction of (**A**) the construction of the SPRi sensor with a titanium and gold layer and a polymer foil applied (the thicknesses of the titanium and gold layers are shown schematically), (**B**) biological layers created during antibody immobilization and subsequent attachment of the analyte contained in the tested sample.

The measurement chips used are made of BK7 glass, but to facilitate gold adsorption (50 nm), a 1 nm titanium layer is placed between the gold and the glass. The structure of the measurement chip is shown in Figure 2A. To functionalize the surface, the chip is immersed in a 1 mM alcoholic solution of 11-mercaptoundecanoic acid for approximately 24 h. After this time, the chip is rinsed twice in ethyl alcohol and water and dried in an argon stream. The next step is to apply a propylene foil, which separates nine measurement sites on a single chip. This allows up to nine samples to be measured in a single measurement. The foil prevents mixing of the solutions. There is glue on the foil that allows it to be attached to the chip.

All procedures for anchoring the antibody to the thiol surface are performed in the next step. The EDC and NHS mixture is applied to the measurement sites for 10 min. During this time, EDC converts the thiol carboxyl group into an ester group, while NHS forms an active, short-lived NHS-ester, as shown in Figure 2B. The peristaltic pump removes excess mixture, and 3 µL of the appropriate antibody is applied from a pipette. The NHS-ester forms a covalent amide bond with the antibody’s amino groups. After another 10 min, excess solutions are removed using a peristaltic pump, and ethanolamine is applied to the active sites to prevent nonspecific adsorption. The antibody immobilization sites are then washed with PBS buffer, and the prepared chip is placed on the prism of the SPRi device using immersion oil. The appropriate angle is selected, and images are taken. The final step in the analysis is the application of the analyte to the measurement sites. After the 10 min association period and a buffer wash, another series of measurements is performed.

## 3. Experimental

### 3.1. Selection of the Appropriate Ligand–Antibody Concentration. Calculation of the Surface Ligand Concentration

**Figure 3:**
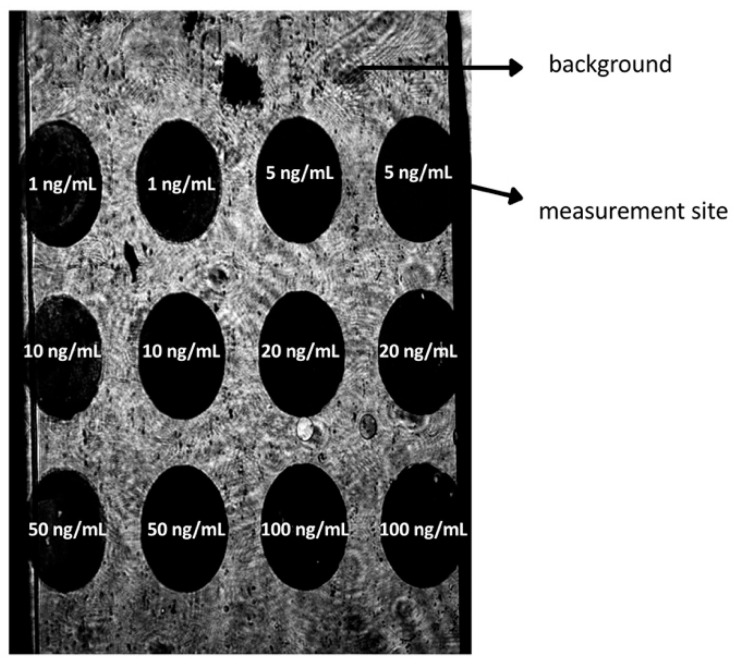
Distribution of the tested concentrations of antibody on the measurement chip. The analytical signal difference in the signal from the antibody at concentrations 1, 5, 10, 20, 50, 100 ng/mL and 11-MUA. Image acquired at the optimal viewing angle, maximizing signal-to-background contrast at the active site.

Each surface on the sensor has a saturation maximum for a given reagent. Determining the appropriate receptor layer concentration enables accurate measurement. To obtain the relationship between the obtained SPRi signal and the antibody concentration, the following experiment was performed: a plate with a gold layer, a polymer foil, and a thiol (11-MUA) layer was placed on the SPRi device prism, and data acquisition was performed at the best SPR angle—the one at which the highest contrast between the background and measurement sites is observed (Figure 3).

**Figure 4:**
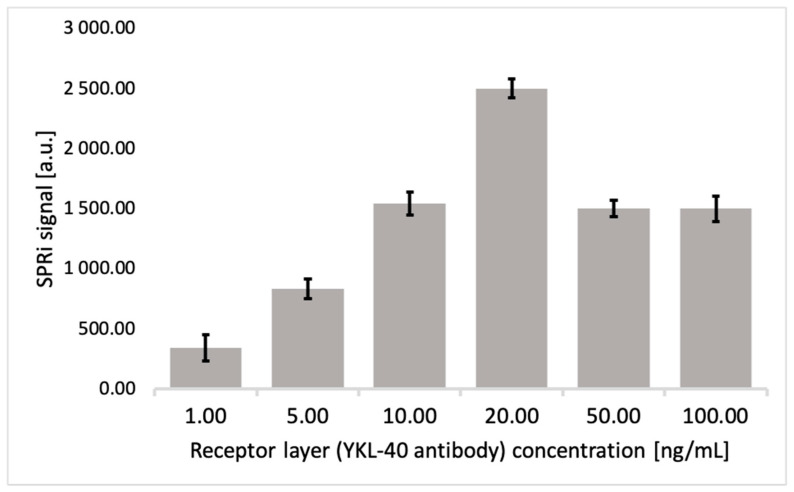
Bar graph showing saturation of the thiol layer with YKL-40-specific antibody. The optimal concentration is 20 ng/mL, pH = 7.4 (Γ = 1.89 × 10^−14^ mol/mm^2^, 1.14 × 10^10^ particles/mm^2^). Error bars indicate the standard deviation calculated from four measurements for each sample.

To ensure that the best angle was selected, acquisition was performed within ±0.2 of the angle chosen, so that five measurements were taken of each 11-MUA layer site. Standard antibody solutions in PBS at concentrations of 1.00, 5.00, 10.00, 20.00, 50.00, and 100.00 ng/mL were then applied to the chip. Each concentration was measured twice on the chip, and the measurement was then repeated after regenerating the sensor surface. This resulted in four SPRi signals for each concentration. After 10 min, the appropriate association time, data were acquired in the same range. The SPRi signal intensities depicted in the graph in Figure 4 are averages of four technical replicates. We observe an increase in the SPRi signal up to a concentration of 20 ng/mL, followed by a decrease until a plateau is reached. Above 20 ng/mL, we no longer observe receptor binding on the 11-MUA surface. The signal drop is caused by high antibody density and reduced accessibility of binding sites. Molecules cannot reach the active sites, binding is limited, and the SPRi signal effectively decreases. We observed an increase in signal with increasing antibody concentration used for surface functionalization up to 20 ng/mL; above this value the signal decreased and stabilized (plateau formation), which we interpret as indicating saturation of available binding sites on the sensor surface. Therefore, 20 ng/mL was deemed optimal because it provides a maximum signal with minimal antibody consumption. It should be noted that higher levels of immobilized ligand do not continue to improve the sensor layer—too high a density can lead to reduced antibody activity due to incorrect orientation, aggregation, or steric effects limiting analyte access to binding sites. To minimize these potential limitations, we used ethanolamine, which blocks residual active sites to reduce background and improve specificity.

The next step was to calculate the surface concentration, which permits control of the functional quality of the surface, as well as normalization of the results and the obtaining of comparable of measurements. The surface concentration was calculated for three independent measurement plates, where the obtained saturation curves were similar to the saturation curve in Figure 4, so that the antibody concentration was assumed to be 20 ng/mL. In the first step, the surface area of one active site was calculated using the circle area formula (*A* = π*r*^2^), where *r* is the radius of the active site (Figure 2), here equal to 1.5 mm. Next, the surface concentration Γ = *n*/*A* (where *n* is the number of moles of antibody and *A* is the surface area of the active site) was calculated from the antibody concentration in solution. The calculations yielded a surface concentration of 1.89 × 10^−14^ mol/mm^2^. This is a fairly typical value for the receptor layer in this SPRi technique. It corresponds to approximately 1.14 × 10^10^ particles/mm^2^, indicating a densely distributed antibody layer without crowding.

### 3.2. Selection of Rinse Frequency

**Figure 5:**
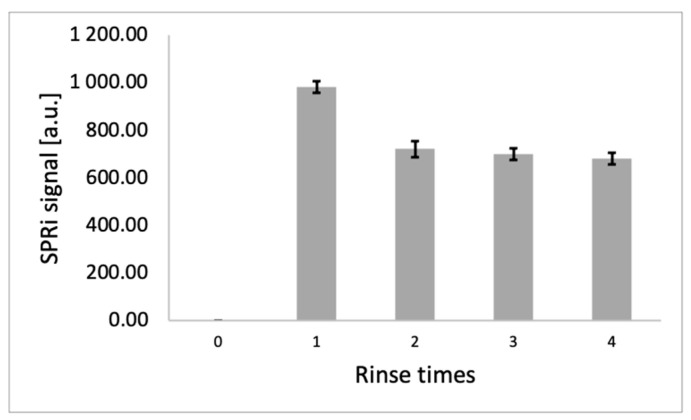
Dependence of the SPRi signal on the number of washes. YKL-40 protein concentration = 10 ng/mL, Γ = 1.89 × 10^−14^ mol/mm^2^, pH = 7.4. The error bars shown in Figure represent the standard deviation from 4 independent replicates.

Selecting the rinse frequency prevents washing of the desired adsorbed molecules from the layers. This allows us to determine whether they are immobilized on the surface despite the use of PBS buffer. Washing is an essential step that removes unbound molecules, which may interfere with the final analytical result, from the sample matrix. To establish whether washing disrupts the structure of the adsorbed layers, the resulting SPRi signal is compared. If its value decreases or increases after washing, we observe an adverse effect of the PBS buffer on the deposited layers. To investigate the effect of rinsing on the signal obtained, an antibody was immobilized on the surface of a gold chip at Γ= 1.89 × 10^−14^ mol/mm^2^. After completing this procedure, data acquisition for the antibody layer was performed. A standard YKL-40 solution at a concentration of 10 ng/mL was then applied to the chip at each measurement site of the sensor. After 10 min, the excess YKL-40 solution was removed from the chip surface, and the chip was washed once with PBS buffer. The measurement was then performed. These steps were repeated for four rinses of the measurement surfaces. The results obtained are presented in Figure 5. The error bars shown in the figure represent the standard deviation from four independent replicates. The low standard deviations confirm the repeatability of the results (mean SD = 20.90 a.u.).

According to the curve, we observe that as the wash number increases, the signals begin to decrease—this indicates the unfavorable effect of a large number of washes on the measurement. This may indicate an undesirable dissociation of the analyte molecules bound to the receptor surface. We also observe that the signal decrease does not exceed 30% of the best signal, indicating layer stability and good protein–antibody affinity, and lying within the range of typical reported deviations in SPRi studies [37]. A single wash was chosen as the preferred method, and was performed until the end of the experiment to avoid altering the measurement conditions.

### 3.3. Model SPR Curves—Evidence Confirming the Formation of Layers on the Surface of the Gold Plate

Data were acquired for the thiol layers—11-MUA, a YKL-40-specific antibody, and the YKL-40 protein—to verify whether the layers were immobilized on the sensor surface at different ranges depending on the minimum signal. The angles were within a range of 33 to 40 degrees. Each layer should have a minimum at a different SPR angle, with a tendency to present higher angles than the preceding layer.

**Figure 6:**
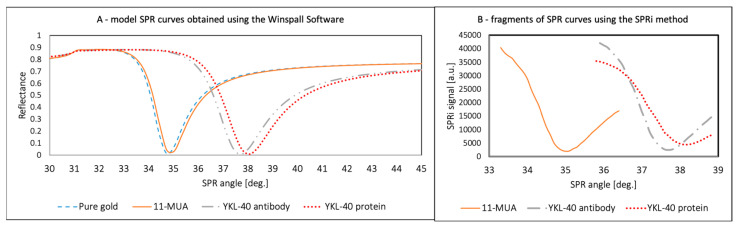
Model SPR curves for each layer forming the biosensor (**A**) and experimental results of the SPR curve minima in different ranges (**B**).

The chip with the 11-MUA layer was placed on an SPRi prism, and data acquisition was performed. Next, the antibody (1000 ng/mL) was immobilized using EDC/NHS chemistry, and ethanolamine was applied to prevent nonspecific adsorption. After rinsing with PBS buffer, another measurement was taken. A 1000 ng/mL protein standard solution was applied, and another measurement was performed. ImageJ software (version 1.53, National Institutes of Health, NIH) was used to obtain SPR signals from the images. Excess standard solutions were used for the studies; the excess provides stability and allows results to be obtained over a wide angular range. The SPR curves for each experimental layer are shown in Figure 6B. Modeling with WinSpall software (version 3.02, Res-Tec, Rosenheim, Germany) was also used to confirm that the experimentally determined minima coincide with the model SPR curves for the given layers (A). In both cases, each curve is shifted toward higher angles relative to the preceding layers, and the minima for the model (A) and the experiment (B) lie in similar ranges. This confirms the formation of layers and the correctness of the experiment.

### 3.4. Analytical Response Study of the Biosensor: Limit of Detection and Quantification

**Figure 7:**
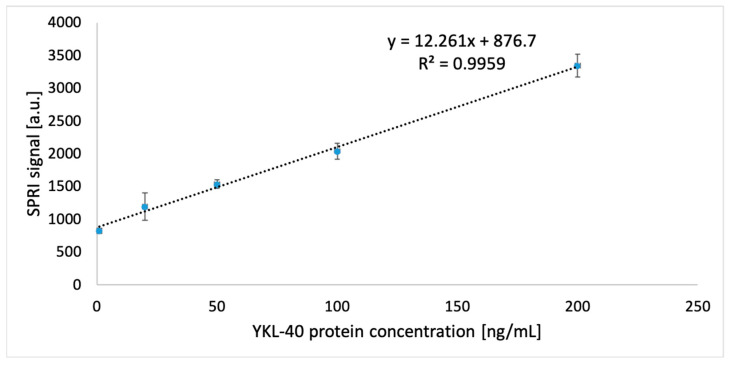
Linear range of calibration curve for YKL-40 protein. Receptor layer concentration = 20 ng/mL, pH = 7.4. Each concentration was tested four times, and the result is the arithmetic mean. Error bars are added based on the calculated standard deviation (SD).

By investigating a sensor’s analytical response, it is possible to determine the linearity range. It is assessed to what extent the relationship is a proportional one. This is important for measuring in further stages of experimentation. Thus, the biosensor’s response is used for calibration, obtaining a relationship between the signal and the concentration of the analyte of interest to the researcher. To examine this dependence in the design of the sensor for YKL-40, a specific antibody to YKL-40 with a concentration of 20 ng/mL was immobilized on the gold and thiol 11-MUA surface using EDC/NHS. Ethanolamine was then used to avoid nonspecific adsorption. Data acquisition at the best angle was made for the antibody layer, and then after 10 min for YKL-40 proteins from standard solutions with 1 to 1000 ng/mL concentrations. Linear dependence was obtained for a range of 1 to 200 ng/mL. The straight part of the calibration curve is shown in Figure 7. The curve equation was also determined, allowing concentrations to be determined in measurements of natural sample signals.

During the experiment, the limits of detection and quantification were also determined using the formulas LOD = (3.3 × SD)/*a* and LOQ = (10 × SD)/*a*, where SD is the standard deviation and *a* is the slope of the simple calibration curve. The LOD value obtained was 0.002 ng/mL, and the LOQ was 0.007 ng/mL. The LOD results were compared with available literature data and are presented in Table 1. Comparison of the detection limit is essential, as it is a significant parameter of analytical performance. It makes it possible to assess whether the method is competitive with existing solutions and whether it has potential for diagnostic applications. Representative analytical methods previously used to determine YKL-40 for which detection limit data were available were selected for comparison. The comparison includes various approaches, to highlight the sensitivity range of the SPRi biosensor under construction.

### 3.5. Precision and Accuracy of the Method

Precision and accuracy are among many validation parameters determined during the development of a new analytical method. Precision describes the degree of agreement between individual analytical results during repeated, independent determinations of a homogeneous sample. Accuracy describes the agreement between the actual value and the value resulting from the analysis. Table 2 presents the results of the precision and accuracy tests. The antibody layers immobilized on an 11-MUA gold chip were prepared according to the antibody immobilization protocol to obtain the given concentration values. Data were then acquired for the receptor layer, and the appropriate concentrations were applied to the surface: the LOQ value of 0.007 ng/mL, the two midpoints of the calibration curve—10 and 100 ng/mL—and the final concentration value of the linear relationship–200 ng/mL. Each measurement was performed four times in independent analyses.

### 3.6. Repeatability and Selectivity

Selectivity is the ability to detect a test substance in the presence of components that may be present in the sample. Three angiogenic proteins, IL-1β, ANG-2, and HIF-1α, were selected as interfering proteins. These are involved in pathological angiogenesis [39]. Due to the many theories concerning the pathology of Alzheimer’s disease, the literature provides information on the involvement of angiogenesis in the progressive destruction of neurons in AD. Large populations of endothelial cells are activated by angiogenesis due to cerebral hypoxia and inflammation. The endothelium secretes a precursor substrate for β-amyloid plaques, contributing to deposition in the brains of AD patients [40]. YKL-40 also has angiogenic potential, as mentioned in the Introduction. It is believed that YKL-40 may participate in cell survival and growth during angiogenesis [41]. Selected interferents also include the total tau and ptau-181 proteins. YKL-40 levels in the cerebrospinal fluid significantly correlate with markers of Alzheimer’s disease, including ptau-181 and total tau, making these proteins relevant interferents in the context of neurodegenerative biomarkers [42]. To test the selectivity of the developed method, 10 solutions were prepared in which each interferent was mixed in a 1:10 and 1:100 concentration ratio with YKL-40 protein. The analysis was performed according to the antibody immobilization protocol described in Section 2.4. After data acquisition for the receptor layer, the protein and interferent mixture was applied to the measurement sites. After 10 min of rinsing with PBS buffer, further measurements of the analyte layer were performed. The concentration of the standard solution YKL-40 was 5 ng/mL, which is the desired concentration for the determination. Each measurement was repeated three times, and the results are the average of the obtained concentrations. They are presented in Table 3. The average recovery from all measurements was 105.19%. The presence of interfering agents does not significantly affect the SPRi signal because the interfering molecules have different epitopes than YKL-40, and therefore do not bind to the receptor layer used. Despite elevated concentrations of interfering agents such as ptau-181 and total tau, which do not occur physiologically, the tests showed no significant SPRi response in their presence.

The method’s repeatability was assessed by measuring a real sample of blood plasma from an AD patient five times. The sample was diluted two-fold. The CV for the obtained results was 4.995%, indicating good repeatability of the SPRI biosensor-based method.

### 3.7. Stability of the Biosensor

A biosensor’s stability is tested because it is one of the key parameters determining its practical usefulness. It indicates the biosensor’s ability to maintain its properties over time. To test biosensor stability, a sample of a standard YKL-40 solution at a concentration of 80 ng/mL and a natural sample of known concentration, determined during a repeatability study, were analyzed, where the concentration was 314.18 ng/mL. All samples used in the experiments were collected, and standard solutions were prepared, aliquoted, and frozen at −80 °C. They were thawed immediately before SPRi analysis. Therefore, these conditions were also met for the reference sample in Table 4, which was not subjected to storage changes. The analysis results are presented in Table 4. The samples were tested twice in independent measurements under each of the following storage conditions:

− 24 h at room temperature,
− 2 h at room temperature,
− 2 h in a refrigerator,
− four freeze–thaw cycles.

The stability parameter is the error Δ [%], which was calculated using the formula: Δ [%] = ((*C*_0_ − *C_i_*)/(*C*_0_ + *C_i_*)) × 200, where *C*_0_ is the control sample concentration (the concentration of a sample not exposed to factors potentially affecting sample stability), and *C_i_* is the determined concentration.

### 3.8. Determination YKL-40 in Real Samples

The biosensor’s utility was confirmed by analyzing natural samples—plasma from actual Alzheimer’s disease patients—and two control groups: plasma from patients with prostatitis and plasma from smokers. The biosensor was prepared according to a protocol involving antibody immobilization at a concentration of Γ = 1.89 × 10^−14^ mol/mm^2^, followed by measurements of the receptor–antibody layer and analyte application. All samples were diluted two-fold to fall within the linear concentration range of the biosensor. The results obtained are presented in Table A1 in Appendix A.

## 4. Discussion and Statistical Analysis

Any sensor design requires prior preparation of the receptor layer. In this case, saturation curves were generated, and then the surface concentration at the measurement site was calculated; its value was Γ = 1.89 × 10^−14^ mol/mm^2^. This prepared layer was used for validation of the analytical method and the SPRi matrix biosensors. The formation of the biosensor layers was also confirmed by testing over a wide angular range, and the appropriate rinsing frequency was selected, proving that a single rinsing of the measurement sites with PBS buffer was the most efficient for our analytical process. The first validation parameter concerned the analytical response of the biosensor, verifying whether a linear response was obtained. The experiment enabled a dynamic range from 1 to 200 ng/mL to be obtained. The limits of detection and quantification were also obtained, namely LOD = 0.002 ng/mL and LOQ = 0.007 ng/mL. These results were compared with those obtained in other studies on the determination of YKL-40. In a study by W. Chaocharoen et al., the electrochemical biosensor developed had a detection limit of 0.07 ng/mL, which the authors describe as significantly lower than that of ELISA tests, making it a promising alternative [23]. In a study by M. Schmalenberg et al., the detection limit of the fluorescent magnetic bead sandwich test sensor was 2.9 ng/mL, which is also significantly higher than that obtained with the SPRi biosensor method in our work [38]. The two previously described literature reports on YKL-40 assays also indicate higher LODs than those obtained in our study, namely 0.014 ng/mL [24] and 0.33 ng/mL [25]. This study confirms that the constructed sensor can operate at low concentrations and in better ranges than the ELISA test. The low standard deviations and CV values indicate that the method is precise and accurate.

Furthermore, the broad sample matrix does not pose a problem for detection, as is confirmed by the selectivity test. The experiment demonstrated that despite the presence of potent interfering agents, the desired concentration could be accurately determined. The repeatability test showed that it was possible to repeat this determination in independent measurements and obtain very similar concentrations, while maintaining sensor stability, which was also confirmed by the test. Exposing the sample to different conditions and a series of freezing and thawing cycles did not significantly affect the determination.

**Figure 8:**
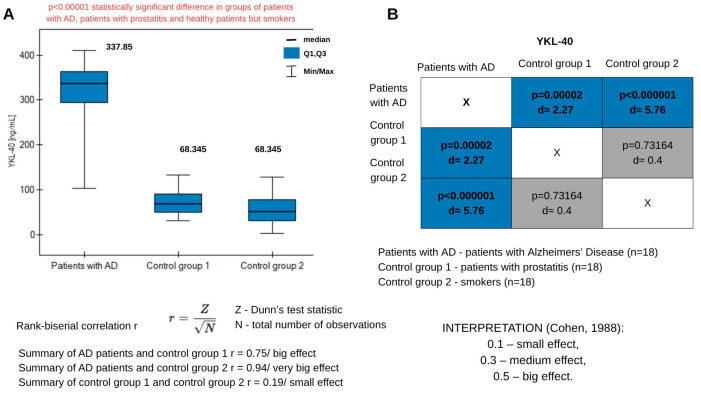
(**A**) graph of changes YKL-40 concentrations in plasma samples from patients with AD and two control groups. Information about the medians is placed above the box plots. (**B**) Dunn–Bonferroni POST-HOC graphical analysis. The boxes contain the p-parameter values and Cohen’s d values.

The most crucial step in testing the sensor’s performance in practice was the determination of YKL-40 in plasma samples from AD patients and control groups. Two control groups were selected for the study: plasma samples from healthy, smoking patients, and a group of plasma samples from patients with prostatitis, to determine whether, despite the inflammation occurring in the body, YKL-40 would exhibit higher concentrations in prostatitis. It is already known that YKL-40 levels can be elevated in various conditions, not only cancer and neurodegenerative diseases. Because elevated YKL-40 levels are observed in inflammation, we wished to separate the groups to examine whether levels in patients with prostatitis would be higher than in healthy individuals. The mean concentration for AD patients was 322.58 ± 69.51 ng/mL. One of the samples from AD patients showed lower concentrations than the entire group (Appendix A, Table A1). Due to the small number of samples, we consider this result a reflection of biological heterogeneity in AD. The literature indicates that YKL-40 exhibits high interindividual variability—its levels in AD may even differ several-fold between patients [43]. In some AD patients, especially in the early stages, the level of this glycoprotein may still be relatively low, increasing only with disease progression. Genetic factors, such as polymorphisms in the CHI3L1 gene, also influence levels [44]. For the group of patients with prostatitis, the mean concentration was 73.52 ± 29.26 ng/mL. In smokers, the mean concentration was 55.25 ± 35.29 ng/mL. These levels are consistent with results obtained in other studies [36]. This confirms that the assay was performed correctly and provides reliable results. The results were also statistically analyzed. The Shapiro–Wilk test indicated a non-normal distribution (*p* = 0.001575), hence data analysis was performed using nonparametric tests. Kruskal–Wallis ANOVA for studying more than two groups indicated the high statistical significance of the differences in concentrations between the AD group, the healthy control group, and the prostatitis group. The results are presented in Figure 8A. Post Hoc analysis showed that statistical significance in the concentration differences arose precisely between the control and AD groups (Figure 8B). No statistically significant differences were found within the control groups, and so in the subsequent analysis they were combined into a single control group. To further examine the differences, rank-biserial correlation calculations were performed. The results and calculation method are presented in Figure 8. The differences between the AD patients and control group 1 are large and statistically significant. This means that the YKL-40 concentrations differ between the two groups. The effect is even more substantial when comparing the AD patients with control group 2. In practice, the results for these two groups barely overlap. Comparison of the control groups indicates minor differences and a lack of statistical significance. The YKL-40 concentrations in these two groups are similar. Cohen’s d values (Figure 8B) indicate that the AD patient group is clearly distinct from the other two, and the control groups are similar to each other, with differences being small and insignificant.

**Figure 9:**
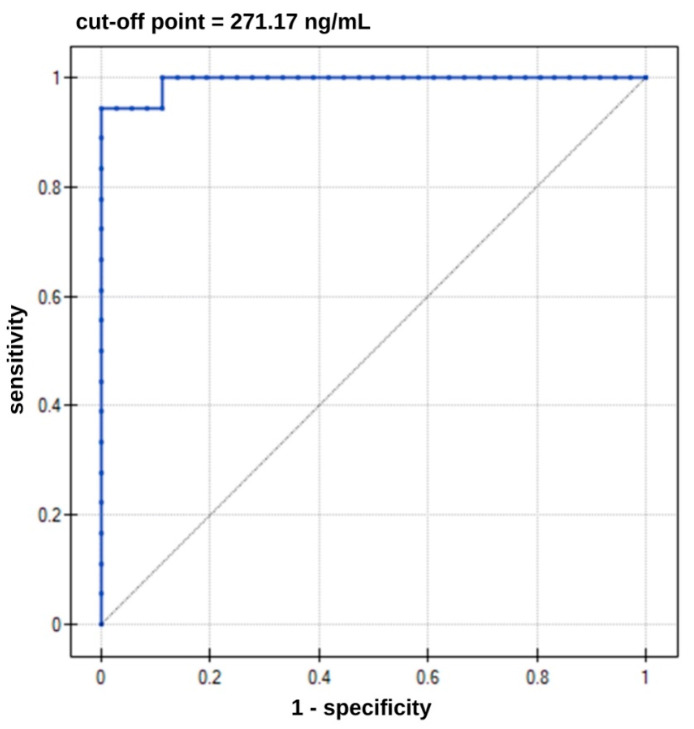
ROC curve for YKL-40 (*n* = 54).

To examine the diagnostic usefulness of the YKL-40 biomarker, ROC curves were performed, which are presented in Figure 9. Essential parameters are listed in Table 5. High statistical significance was obtained for the results.

Increasing concentrations of YKL-40 protein indicate increasing risk of developing AD. Concentrations above the cut-off value of 271.17 ng/mL may indicate the presence of AD. The AUC value close to one suggests that patients were accurately assigned to the disease and control groups. This assignment was not random. Table 5 also provides sensitivity and specificity values. The probability of detecting a healthy individual is 100%. The negative predictive value (NPV) indicates a 97% probability of a person’s not having the disease in the case of a negative test. However, the PPV indicates a 100% probability of having AD in case of a positive test result.

## 5. Conclusions

The constructed SPRi biosensor sensitive to YKL-40 demonstrates good validation parameters, allowing its use in the analysis of biological materials such as blood plasma. Although YKL-40 is not a biomarker specific solely to Alzheimer’s disease, it demonstrates significantly elevated concentrations in patients with this condition. Incorporating YKL-40 into biomarker panels increases diagnostic sensitivity and supports the differentiation of neurodegenerative disorders. Our results indicate that the SPRi biosensor has the potential for use in clinical trials, particularly in multimarker approaches, where it can be a valuable complement to existing diagnostic tools.

## Figures and Tables

**Table 1:** Detection limit values obtained by different methods.

LOD	Detection Method
0.002 ng/mL	SPRi method (this article)
0.07 ng/mL [23]	Electrochemical sensor using polyclonal antibodies immobilized on gold electrodes, an electrochemical flow system with capacitive signal generation [23]
0.014 ng/mL [24]	Photothermal YKL-40 screening platform by coupling platinum nanoparticles (PtNPs) excited by near-infrared (NIR) light with a handheld digital thermometer, using an antibody as a receptor layer [24]
0.33 ng/mL [25]	Surface Plasmon Resonance (SPR) using a commercially available BIAcore 2000 device with antibodies in the receptor layer [25]
2.9 ng/mL [38]	Fluorescent immunoenzymatic assay using magnetic beads [38]

**Table 2:** Precision and accuracy parameters of the tested method. Each concentration was analyzed four times (n = 4, SD—standard deviation, CV—coefficient of variation).

Standard Solution Concentration [ng/mL]	Mean Concentration from 4 Analysis [ng/mL]	SD [ng/mL]	CV [%]
0.007	0.008	0.0009	0.12
10.00	11.21	1.04	9.24
100.00	103.26	3.95	3.83
200.00	200.98	7.02	3.49

**Table 3:** Selectivity of the tested method. Each concentration was analyzed three times (*n* = 3). The concentration of the standard solution was 5 ng/mL, which is the desired concentration for the determination.

Interferent	Concentration Ratio	Mean Concentration Marked [ng/mL]	Recovery [%]
HIF-1α	1:100	5.24	104.71
	1:10	5.26	105.26
ANG-2	1:100	5.26	105.22
	1:10	4.62	92.49
IL-1β	1:100	5.43	108.63
	1:10	5.21	104.26
Total tau	1:100	5.83	116.52
	1:10	5.64	112.82
Ptau-181	1:100	5.02	100.48
	1:100	5.07	101.48

**Table 4:** Stability of the biosensor.

Concentration of Control Sample C_0_ [ng/mL]	Analyte Storage Conditions	Concentration Marked C_i_ [ng/mL]	Δ [%]
80	24 h at room temperature	98.45	17.38
		92.12	
	2 h at room temperature	81.23	2.07
		82.12	
	2 h in a refrigerator	80.23	0.81
		81.07	
	four freeze–thaw cycles	81.78	1.17
		80.11	
314.18	24 h at room temperature	320.42	1.61
		319.34	
	2 h at room temperature	315.16	0.09
		314.99	
	2 h in a refrigerator	315.78	0.49
		316.89	
	four freeze–thaw cycles	321.11	1.64
		318.87	

**Table 5:** The data characterizing the ROC analysis for YKL-40. AUC—Area Under Curve, PPV—Positive Predictive Value, NPV—Negative Predictive Value, CI = 95%.

Direction of the Diagnostic Variable	AUC	Sensitivity [%]	Specificity [%]	PPV [%]	NPV [%]	Cut-Off Point	*p*-Value
stimulant	0.99	94.00	100.00	100.00	97.00	271.17 ng/mL	<0.000001

## Data Availability

Data available on request from the authors.

## Appendix A

**Table A1:** YKL-40 assay results in different sample groups.

Sample Number	Concentration of YKL-40 in Plasma Samples from Patients with Alzheimer Disease [ng/mL]	Concentration of YKL-40 in Plasma Samples in Control Group Patients with Prostatitis [ng/mL]	Concentration of YKL-40 in Plasma Samples in Control Group Smokers [ng/mL]
1	300.29	79.81	30.24
2	386.88	39.87	65.85
3	335.65	102.49	2.46
4	357.22	87.67	15.53
5	271.17	48.44	10.91
6	397.56	30.43	110.33
7	291.73	94.47	60.05
8	365.65	69.82	128.39
9	303.45	66.87	33.38
10	338.52	43.24	47.32
11	307.17	91.59	40.35
12	281.46	41.43	93.85
13	345.19	64.73	69.59
14	410.36	87.24	91.33
15	386.70	54.69	24.19
16	102.60	127.43	36.34
17	284.16	131.92	54.54
18	340.61	61.13	79.92

## References

[1] Tizaoui K., Yang J.W., Lee K.H., Kim J.H., Kim M., Yoon S., Jung Y., Park J.B., An K., Choi H. (2022). The role of YKL-40 in the pathogenesis of autoimmune diseases: A comprehensive review. Int. J. Biol. Sci..

[2] Mavroudis I., Chowdhury R., Petridis F., Karantali E., Chatzikonstantinou S., Balmus I.M., Luca I.S., Ciobica A., Kazis D. (2021). YKL-40 as a Potential Biomarker for the Differential Diagnosis of Alzheimer’s Disease. Medicina.

[3] Faibish M., Francescone R., Bentley B., Yan W., Shao R. (2011). A YKL-40-neutralizing antibody blocks tumor angiogenesis and progression: A potential therapeutic agent in cancers. Mol. Cancer Ther..

[4] Johansen J.S., Høyer P.E., Larsen L.A., Price P.A., Møllgård K. (2007). YKL-40 Protein Expression in the Early Developing Human Musculoskeletal System. J. Histochem. Cytochem..

[5] Turkyilmaz A., Devrimsel G., Kirbas A., Cicek Y., Karkucak M., Capkin E. (2013). Gokmen, F. Relationship between pulse wave velocity and serum YKL-40 level in patients with early rheumatoid arthritis. Rheumatol. Int..

[6] Salomon J., Matusiak Ł., Nowicka-Suszko D., Szepietowski J. (2018). Chitinase-3-like protein 1 (YKL-40) is a biomarker of severity of joint involvement in psoriatic arthritis. Adv. Dermatol. Allergol..

[7] Peltomaa R., Paimela L., Harvey S., Helve T., Leirisalo-Repo M. (2001). Increased level of YKL-40 in sera from patients with early rheumatoid arthritis: A new marker for disease activity. Rheumatol. Int..

[8] Wang Y., Wong C.W., Yan M., Li L., Liu T., Mei-Yu Or P., Kwok-Wing Tsui S., Miu-Yee Waye M., Man-Lok Chan A. (2018). Differential regulation of the pro-inflammatory biomarker, YKL-40/CHI3L1, by PTEN/Phosphoinositide 3-kinase and JAK2/STAT3 pathways in glioblastoma. Cancer Lett..

[9] Wang Z., Wang S., Jia Z., Hu Y., Cao D., Yang M., Liu L., Gao L., Qiu S., Yan W. (2023). YKL-40 derived from infiltrating macrophages cooperates with GDF15 to establish an immune suppressive microenvironment in gallbladder cancer. Cancer Lett..

[10] Chen Y., Zhang S., Wang Q., Zhang X. (2017). Tumor-recruited M2 macrophages promote gastric and breast cancer metastasis via M2 macrophage-secreted CHI3L1 protein. J. Hematol. Oncol..

[11] Zhao T., Zeng J., Xu Y., Su Z., Chong Y., Ling T., Xu H., Shi H., Zhu M., Mo Q. (2022). Chitinase-3 like-protein-1 promotes glioma progression via the NF-κB signaling pathway and tumor microenvironment reprogramming. Theranostics.

[12] Akiyama H., Barger S., Barnum S., Bradt B., Bauer J., Cole G.M., Cooper N.R., Eikelenboom P., Emmerling M., Fiebich B.L. (2010). Inflammation and Alzheimer’s disease. Neurobiol. Aging.

[13] Selkoe D.J. (2011). Alzheimer’s disease: Genes, proteins, and therapy. Physiol. Rev..

[14] Blennow K., Dubois B., Fagan A.M., Lewczuk P., de Leon M.J., Hampel H. (2015). Clinical utility of cerebrospinal fluid biomarkers in the diagnosis of early Alzheimer’s disease. Alzheimer’s Dement..

[15] Johnson L.V., Leitner W.P., Rivest A.J., Staples M.K., Radeke M.J., Anderson D.H. (2002). The Alzheimer’s A beta-peptide is deposited at sites of complement activation in pathologic deposits associated with aging and age-related macular degeneration. Proc. Natl. Acad. Sci. USA.

[16] Bonneh-Barkay D., Bissel S.J., Wang G., Fish K.N., Nicholl G.C., Darko S.W., Medina-Flores R., Murphey-Corb M., Rajakumar P.A., Nyaundi J. (2008). YKL-40, a Marker of Simian Immunodeficiency Virus Encephalitis, Modulates the Biological Activity of Basic Fibroblast Growth Factor. Am. J. Pathol..

[17] Bonneh-Barkay D., Wang G., Starkey A., Hamilton R.L., A Wiley C. (2010). In vivo CHI3L1 (YKL-40) expression in astrocytes in acute and chronic neurological diseases. J. Neuroinflamm..

[18] Bonneh-Barkay D., Zagadailov P., Zou H., Niyonkuru C., Figley M., Starkey A., Wang G., Bissel S.J., Wiley C.A., Wagner A.K. (2010). YKL-40 Expression in Traumatic Brain Injury: An Initial Analysis. J. Neurotrauma.

[19] Comabella M., Fernández M., Martin R., Rivera-Vallvé S., Borrás E., Chiva C., Julià E., Rovira A., Cantó E., Alvarez-Cermeño J.C. (2010). Cerebrospinal fluid chitinase 3-like 1 levels are associated with conversion to multiple sclerosis. Brain.

[20] Llorens F., Thüne K., Tahir W., Kanata E., Diaz-Lucena D., Xanthopoulos K., Kovatsi E., Pleschka C., Garcia-Esparcia P., Schmitz M. (2017). YKL-40 in the brain and cerebrospinal fluid of neurodegenerative dementias. Mol. Neurodegener..

[21] Pase M.P., Himali J.J., Puerta R., Beiser A.S., Gonzales M.M., Satizabal C.L., Yang Q., Aparicio H.J., Kojis D.J., Decarli C.S. (2024). Association of Plasma YKL-40 With MRI, CSF, and Cognitive Markers of Brain Health and Dementia. Neurology.

[22] Molinuevo J.L., Ayton S., Batrla R., Bednar M.M., Bittner T., Cummings J., Fagan A.M., Hampel H., Mielke M.M., Mikulskis A. (2018). Current state of Alzheimer’s fluid biomarkers. Acta Neuropathol..

[23] Chaocharoen W., Suginta W., Limbut W., Ranok A., Numnuam A., Khunkaewla P., Kanatharana P., Thavarungkul P., Schulte A. (2015). Electrochemical detection of the disease marker human chitinase-3-like protein 1 by matching antibody-modified gold electrodes as label-free immunosensors. Bioelectrochemistry.

[24] Yu S., Ke Q., Cai F., Gong S., Huang R., Fan C. (2023). Platinum nanozyme-mediated temperature sensor for sensitive photothermal immunoassay of YKL-40 under near-infrared light. Sensors Diagn..

[25] Naglot S., Aggarwal P., Dey S., Dalal K. (2016). Estimation of Serum YKL-40 by Real-Time Surface Plasmon Resonance Technology in North-Indian Asthma Patients. J. Clin. Lab. Anal..

[26] Chirco A., Zielinska Z., Gorodkiewicz E., Meacci E., Margheri G. (2025). Detection of erythropoietin in blood plasma through an SPRi-based biosensor. Talanta.

[27] Xu J., Zhang P., Chen Y. (2024). Surface Plasmon Resonance Biosensors: A Review of Molecular Imaging with High Spatial Resolution. Biosensors.

[28] Kretschm E., Raether H. (2014). Radiative Decay of Non Radiative Surface Plasmons Excited by Light. Z. Für Naturforschung A.

[29] Homola J. (2003). Present and future of surface plasmon resonance biosensors. Anal. Bioanal. Chem..

[30] Scarano S., Scuffi C., Mascini M., Minunni M. (2010). Surface plasmon resonance imaging (SPRi)-based sensing: A new approach in signal sampling and management. Biosens. Bioelectron..

[31] Das S., Devireddy R., Gartia M.R. (2023). Surface Plasmon Resonance (SPR) Sensor for Cancer Biomarker Detection. Biosensors.

[32] Rezabakhsh A., Rahbarghazi R., Fathi F. (2020). Surface plasmon resonance biosensors for detection of Alzheimer’s biomarkers; an effective step in early and accurate diagnosis. Biosens. Bioelectron..

[33] Haleem A., Javaid M., Singh R.P., Suman R., Rab S. (2021). Biosensors applications in medical field: A brief review. Sensors Int..

[34] Wilczyńska K., Waszkiewicz N. (2020). Diagnostic Utility of Selected Serum Dementia Biomarkers: Amyloid β-40, Amyloid β-42, Tau Protein, and YKL-40: A Review. J. Clin. Med..

[35] Craig-Schapiro R., Perrin R.J., Roe C.M., Xiong C., Carter D., Cairns N.J., Mintun M.A., Peskind E.R., Li G., Galasko D.R. (2010). YKL-40: A Novel Prognostic Fluid Biomarker for Preclinical Alzheimer’s Disease. Biol. Psychiatry.

[36] Choi J., Lee H.-W., Suk K. (2011). Plasma level of chitinase 3-like 1 protein increases in patients with early Alzheimer’s disease. J. Neurol..

[37] Zielinska Z., Sankiewicz A., Kalinowska N., Zelazowska-Rutkowska B., Guszcz T., Ambroziak L., Kondratiuk M., Gorodkiewicz E. (2025). Carboxymethyl Dextran-Based Biosensor for Simultaneous Determination of IDO-1 and IFN-Gamma in Biological Material. Biosensors.

[38] Schmalenberg M., Beaudoin C., Bulst L., Steubl D., Luppa P.B. (2015). Magnetic bead fluorescent immunoassay for the rapid detection of the novel inflammation marker YKL40 at the point-of-care. J. Immunol. Methods.

[39] Zielinska Z., Oldak L., Guszcz T., Hermanowicz A., Gorodkiewicz E. (2024). SPRi Biosensor for Simultaneous Determination of HIF-1α, Angiopoietin-2, and Interleukin-1β in Blood Plasma. Sensors.

[40] Vagnucci A.H., Li W.W. (2003). Alzheimer’s disease and angiogenesis. Lancet.

[41] Pouyafar A., Heydarabad M.Z., Mahboob S., Mokhtarzadeh A., Rahbarghazi R. (2018). Angiogenic potential of YKL-40 in the dynamics of tumor niche. Biomed. Pharmacother..

[42] Muszyński P., Groblewska M., Kulczyńska-Przybik A., Kułakowska A., Mroczko B. (2017). YKL-40 as a Potential Biomarker and a Possible Target in Therapeutic Strategies of Alzheimer’s Disease. Curr. Neuropharmacol..

[43] Kester M.I., Teunissen C.E., Sutphen C., Herries E.M., Ladenson J.H., Xiong C., Scheltens P., van der Flier W.M., Morris J.C., Holtzman D.M. (2015). Cerebrospinal fluid VILIP-1 and YKL-40, candidate biomarkers to diagnose, predict and monitor Alzheimer’s disease in a memory clinic cohort. Alzheimer’s Res. Ther..

[44] Deming Y., Black K., Carrell D., Cai Y., Del-Aguila J.L., Fernandez M.V., Budde J., Ma S., Saef B., Howells B. (2016). Chitinase-3-like 1 protein (CHI3L1) locus influences cerebrospinal fluid levels of YKL-40. BMC Neurol..

